# The healthcare inequality among middle-aged and older adults in China: a comparative analysis between the full samples and the homogeneous population

**DOI:** 10.1186/s13561-022-00383-x

**Published:** 2022-06-28

**Authors:** Liping Fu, Ya’nan Fang, Yongqing Dong

**Affiliations:** 1 College of Management and Economics, Tianjin University; Center for Social Science Survey and Data, Tianjin University, Tianjin, 300072 China; 2 College of Politics and Public Administration, Qinghai Minzu University, Qinghai, 810007 China

**Keywords:** Homogeneous population, Inequality, Concentration index, Socioeconomic status

## Abstract

**Background:**

In the Chinese population, the middle-aged and older adults are the two main segments that utilize a large portion of healthcare. With the fast growth of the two segments, the demands of healthcare services increases significantly. The issue related to inequality in utilization of healthcare emerges with the growth and it deserves more attention. Most existing studies discuss overall inequality. Less attention is paid to inequality among subdivisions, that is, relative inequality. This study focuses on the inequality of healthcare utilization among the homogeneous population and the inequality of the full samples in China.

**Methods:**

Data were obtained from four waves of the China Health and Retirement Longitudinal Study (CHARLS): 2011, 2013, 2015 and 2018. First, the Concentration Index (CI) was used to measure the inequality of outpatient, inpatient and preventive care for the samples, and regression analysis was applied to decompose the contributing factors of inequality. Then SOM is introduced to identify homogeneous population through clustering and measure the inequality in three types of healthcare utilization among homogeneous population. Based on this, the difference between absolute inequalities and relative inequalities was discussed.

**Results:**

The preventive care is shown to have the highest degree of inequality inclined to the rich and has the largest increase (CI: 0.048 in 2011 ~ 0.086 in 2018); The inequality degree in outpatient care appears to be the smallest (CI: -0.028 in 2011 ~ 0.014 in 2018). The decomposition results show that age, education, income, chronic disease and self-reported health issues help explain a large portion of inequality in outpatient and inpatient care. And the contribution of socioeconomic factors and education to the inequality of preventive care is the largest. In regards to three types of healthcare among the homogeneous population, the degree of inequality seems to be higher among group with high socioeconomic status than those with lower socioeconomic status. In particular, for the people who are in the high socioeconomic group, the degree of inequality in preventive care is consistently higher than in outpatient and inpatient care. The inequality degree of preventive care in the low socioeconomic status group varies significantly with the flexibility of their response to policies.

**Conclusions:**

Key policy recommendations include establishing a health examination card and continuously improving the fit of free preventive care with the needs of the middle-aged and older adults; developing CCB activities to avoid people’s excessive utilization in the high socioeconomic status group or insufficient utilization in the low socioeconomic status group; reasonable control of reimbursement and out-of-pocket payments.

## Background

Health is a basic right of the citizens [1]. In terms of public policy, equal access to medical resources is a key factor in realizing the right to health [2]. China’s healthcare resources have long faced the issues concerning uneven access and partial distribution, which are manifested in the high-cost of receiving healthcare [3, 4]. It hinders people from pursuing the right to health and causes unequal utilization of healthcare. Achieving health equality and improving the availability and affordability of healthcare are the goals that the Chinese government has been striving for, and it is also the state of healthcare resource supply that people expect. According to recent government reports, the Healthy China Strategy was raised to the level of national priority, and a series of policies were issued as the effort to achieve the goals of “health for all” and “comprehensive health” [5]. It should be noted that, as it is important to address the needs of the overall population, directing special attention to disadvantaged groups which is in low socioeconomic status. It is the key to ensure equality [6] in healthcare services. The elderly, as a group most in need of medical resources, should receive attention. And as China’s aging population grows rapidly, by 2040, the older adults aged 65 and over will exceed 20% of the nation’s population. The early twenty-first century will be the fastest-growing period of China’s population aging [7]. This poses a challenge for the elderly to use healthcare resources equally.

In 2009, China officially launched a policy called the New Health Care Reform (NHCR). The goal of the initiative was to offer safe, effective, convenient and affordable healthcare for the Chinese people. NHCR reforms touch on four aspects of the healthcare system: essential drug management, healthcare services process management, health insurance, and hospital management. Specific measures include an introduction of a zero-markup sales policy for essential drug prices, the establishment of a two-directional referral system, the expansion of health insurance coverage, the unification of the basic health insurance system for urban and rural residents, and the establishment of a public welfare-oriented assessment and evaluation mechanism in hospitals. However, these measures are still ineffective to completely solving the problem of “expensive healthcare”. And as more people are included in the medical insurance plan, demands for healthcare services increased dramatically as a result [8]. During the period between 2010 and 2018, the number of patients increased significantly in three healthcare services. The increased percentages are: outpatient care, 58.6%, inpatient care, 79.6%, and preventative care, 50.2% [9]. However, whether the sharp increases mean that NHCR alleviated the health burden and eased the inequality in the use of medical resources needs to be further investigated.

The measurement of equality in the healthcare field generated a fair amount of research interests, and it is also a subject to which citizens and governments should pay more attention. Related researches are divided into two categories. One is the research on influencing factors. Some researchers found that residential location [10], austerity measures of national health expenditure [11], socioeconomic level [12], expenditure and education [13] have a greater impact on the inequality in healthcare resources utilization. Other scholars found that unequal utilization of healthcare resources can cause differences in health [14] and even affect mortality [15]. Studies in the second category focus on the measurement of inequality. Different types of healthcare resources are measured, such as health inequalities during the COVID-19 pandemic [16], inequality in the use of oral cavity healthcare [17], and inequality in socioeconomic and health financing in maternal mortality [18], etc. The perspective of inequality measurement is threefold: one is to measure the current status [19]; the second is a comparative analysis based on before and after the policy is implemented [20]; the third is based on time series to see the evolution [21]. Most of the above-mentioned studies found that there is clear inequality in healthcare services due to economic inequality which is called absolute inequality. However, few studies considered whether inequality exists among homogeneous people and what its development trend is. Homogeneous groups are groups that are divided based on some indicators and are the same or similar in some respects [22].

The factors in existing studies can be used to measure the economic homogeneity group are income [23], expenditure [24] and living conditions [25]. These factors can all identify the financial capacity to pay for medical expenses. But most studies use only one of the economic indicators to classify healthcare utilization population. Such a single-variable analysis cannot fully measure the financial capacity of an individual to cover medical expenses. The introduction of the concept of homogeneous population in this study may help improve the single-variable-based measurement of the economic differences. And we must admit that healthcare is a limited right, and inequality will definitely exist. Maintaining relative equality and a certain degree of flexibility in the healthcare system is the key to measuring the success of universal healthcare (UHC) [26]. And the world faces multiple health financing challenges as the global health burden evolves. While countries prioritize UHC coverage under the Sustainable Development Goals [27]. However, making the limited capital investment play a greater equality value needs to be paid attention to. Especially, in developing countries, the per capital level of government funding is at a low level. For example, the per capita level of government funding at $25 in Zimbabwe is well below the Chatham House estimated $86 needed to provide an essential benefit package [28]; India is also at comparatively low levels of health spending [29], and the elderly population is costlier to support for their healthcare needs in the future [30]. Thus, for low or middle-income countries, the equity value brought about by limited health spending is more important. And many developing countries are moving towards UHC [31]. So under the circumstance of limited health expenditure, achieving equal utilization of healthcare among homogeneous population proposed in this study is an alternative direction, especially for developing countries that cannot provide high-welfare medical services due to economic development (such as GDP) [30]. And similar studies can be conducted in other countries to identify inequalities among homogeneous population.

This study takes the middle-aged and older adults in China as the research object. First, we measure the inequality in outpatient utilization, inpatient and preventive care based on the samples. Then, self-organizing maps (SOM) were introduced into the analysis combined with the concentration index (CI) method. Based on the socioeconomic level clustering of the samples, CI was used to measure the degree of inequality in the homogeneous population. And then achieve the following three goals: to clarify how the inequality trend of the use of different types of health care among middle-aged and elderly people develop; to explore the factors that affect inequality; to compare the inequality degree among the homogeneous population and clarify its relationship with the inequality degree in the samples.

## Methods

### Data sources

The study sample was drawn from four waves (2011, 2013, 2015 and 2018) of the China Health and Retirement Longitudinal Study (CHARLS). The CHARLS aims to collect a set of high-quality micro-data representing the families and individuals aged 45 and above in China. Its national baseline survey was launched in 2011, covering 150 county-level units, 450 village-level units, and about 17,000 participants in 10,000 households through the PPS sampling method. The questionnaire response rate was 80.51%. Therefore, the sample is nationally representative. And these households were re-surveyed after two or three years. The questionnaire contains demographic background, family structure, employment status, retirement and pension status, household expenditures, and health information.

This study aims to study the changes in the healthcare inequality and contributing factors for the middle-aged and older adults in China, so it is necessary to delete samples with a missing variable. Finally, in four waves, 2,098 samples from 2011 were retained; 4,460 samples from 2013 were retained; 4,449 samples from 2015 were retained; 4,503 samples from 2018 were retained.

### Standardization of healthcare service utilization

Differences in demographic characteristics, economic level, and health status of respondents will directly affect their healthcare services utilization. And the CHARLS questionnaire has time limits when asking about the use of healthcare resources. Indirect standardization of healthcare resource utilization before measuring utilization inequality can better reflect the current status of healthcare resource utilization of the samples. In this paper, the indirectly standardized equation is set as logistic regression by referring to the research of other scholars [32–34] and analyzing the characteristics of the CHARLS sample, as follows:

1 $${\text{logit}}(\frac{{p_{i} }}{{1 - p_{i} }}) = \alpha + \beta \ln income_{i} + \sum\limits_{k} {\gamma_{k} x_{ki} } + \sum\limits_{p} {\delta_{p} z_{pi} } + \varepsilon_{i}$$

In the model, the dependent variable represents the probability of healthcare resource utilization. ln*income* represents the income level of the sample, x_k_ represents the sample healthcare demand variable, that is, variables that affect the medical resources utilization, and z_p_ represents other control variables. α, β, γ, δ represent the coefficients of the corresponding variables, and ε represents the random error term. Using the above equation to obtain the predicted value represents the healthcare services utilization.

### Concentration index and decomposition

This study aims to examine the inequity in the use of healthcare services among groups with similar socioeconomic levels and samples. Thus, the CI and decomposition analysis are carried out on samples and homogeneous groups respectively. For computation, a more convenient formula for the concentration index defines it in terms of the covariance between the health variable and the fractional rank in the living standards distribution [35–38]. Where h_i_ is the health sector variable, µ is its mean, and r_i_ = i/N is the fractional rank of individual i in the living standards distribution, with i = 1 for the poorest and i = N for the richest. The index is bounded between -1 and 1, with an index of 0 equivalent to perfect equality. A positive C signifies that a health or healthcare variable is more concentrated among the richer populations and vice versa [39].

2 $${\text{C}} = \frac{2}{\mu }{\text{cov}}(h,r)$$

Next, we will explain how such inequality can be explained through the decomposition of the CI. Wagstaff demonstrates that the health CI can be decomposed into the contributions of individual factors to income-related health inequality, in which each contribution is the product of the sensitivity of heath with respect to that factor and the degree of income-related inequality in that factor [40]. It can be calculated by the linear additive regression model, the formula is as follows:

3 $${\text{y}} = \alpha + \sum\nolimits_{k} {\beta_{k} x_{k} + \varepsilon }$$

the concentration index for y, C, can be written as follows:

4 $${\text{C}} = \sum\nolimits_{{\text{k}}} {(\beta_{k} } \overline{x}_{k} /\mu )C_{k} + GC_{\varepsilon } /\mu$$

where µ is the mean of y, $\overline{x}_{{\text{k}}}$ is the mean of $x{}_{k}$, C_k_ is the concentration index for $x_{k}$(defined analogously to C), and GC_ε_ is the generalized concentration index for the error term (ε). Eq. (4) shows that C is equal to a weighted sum of the concentration indices of the k regressors, where the weight for $x_{k}$ is the elasticity of y with respect to $x_{k} (\eta_{k} = \beta_{k} \frac{{\overline{x}_{k} }}{\mu })$. The residual component captured by the last term reflects the income-related inequality in health that is not explained by systematic variation in the regressors by income, which should approach zero for a well-specified model. Then we calculated the percentage contribution of each regressors (100Qk /C). Of note, the negative and positive contributions may cancel out in the aggregate and the percentage contribution of the regressors and error term sum would be 100%, so the percentage contribution of several regressors may represent large positive and negative contributions, even over 100%.

Inequalities in health or healthcare are associated with demographic factors, health state, economic level, insurance, medical distance and work state. This study will analyze the contribution of inequality through these 6 types of variables. Coindex command in STATA 15.0 was used to calculate the CI. Then the factor specific elasticity, concentration indices, and contributions in Eq. (4) can be computed and displayed with the loop statement.

### Self-organizing maps

Relying on basic descriptors of wealth such as median household income does not always provide sufficient nuance when disaggregating the economic levels of groups [41]. To evaluate the economic level of the groups more comprehensively, we employ an unsupervised machine learning clustering technique, the self-organizing map (SOM) [42]. SOM algorithms were applied to a wide range of disciplines [43]. In recent years, it became more and more mature in the field of management. And some scholars successfully applied the SOM method to determine the economic level of the population through socioeconomic characteristics [44, 45]. But they only cluster income. This article will use multi-factor clustering to measure people’s economic levels. The SOM algorithm used in this study is adapted from the latest MATLAB-based SOM toolbox.

Because the sample income of the questionnaire survey may be false or incorrect in CHARLS. To classify the socioeconomic level of the samples more accurately, we first select the factors that have a greater impact on the socioeconomic level. Income as a direct measure of the level of wealth will be used as one of the measuring factors. Household expenditure can measure the economic level of the sample, and it has a greater impact on the use of medical resources [12]. However, the sample in this paper is an individual, so the average annual household expenditure is used to measure the personal economic level. The installation or improvement of rural households’ environmental sanitation facilities depends on factors such as their socioeconomic status, payment costs, knowledge, attitudes, and hygiene behaviors [46]. And some of the CHARLS samples are concentrated in rural areas, so six household facilities such as toilet flushing, electricity use, running water, bath facilities, natural gas supply, and broadband internet connection are considered as one of the economic level influencing factors. To ensure the balance of data, each facility is assigned a value of 100. Each item owned by the sample could increase the household facilities level by 100. Finally, to avoid the influence of the dimensions of different factors on the data analysis, the three factors are standardized.

**Fig. 1:**
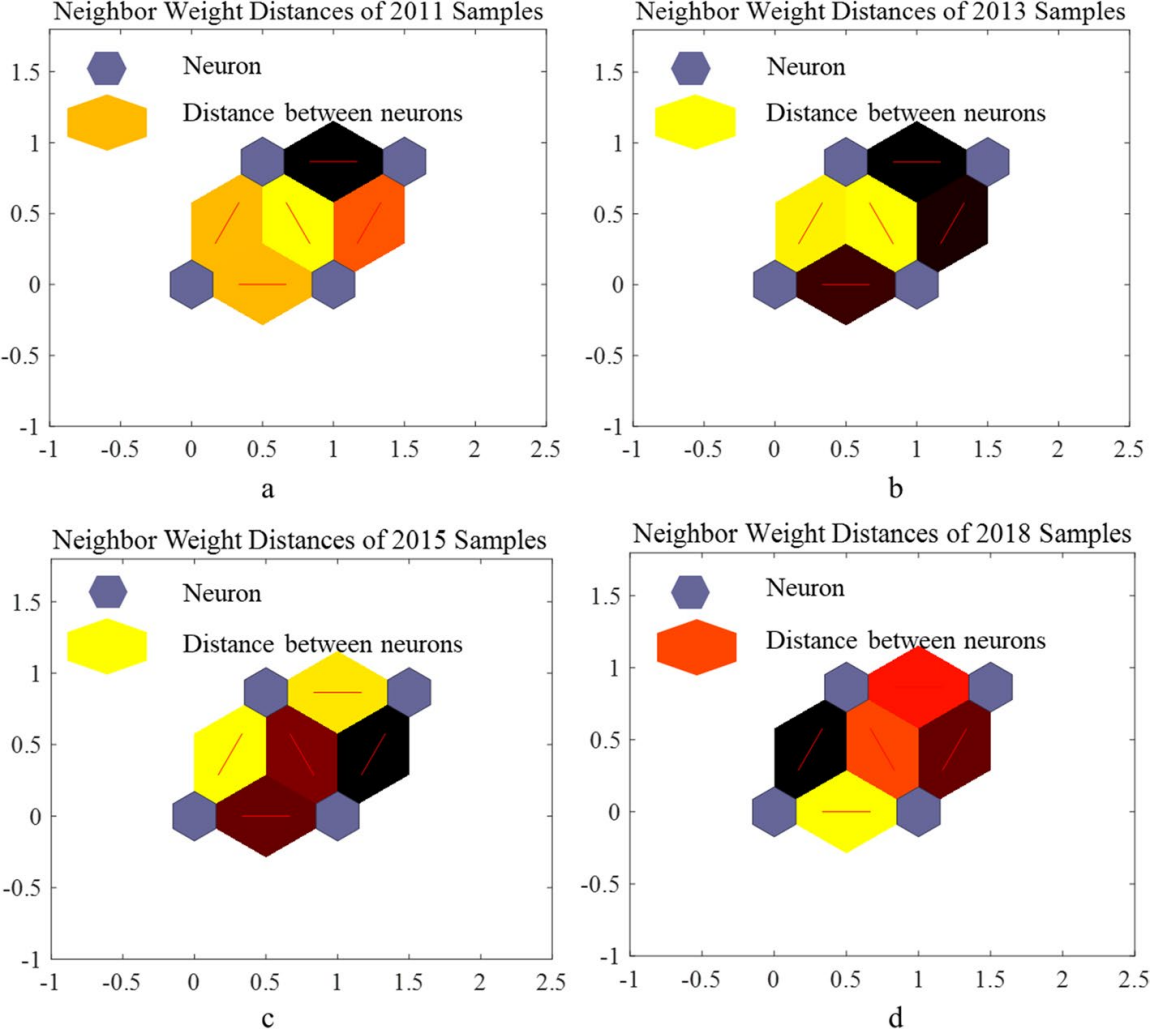
Neurons distances of four waves

We trained the SOM separately for every year’s sample, because the samples will be deleted or added. The 2*2 matrix is set, because the samples only need to be divided into two categories. The neuron numbers are numbered from the left side of the bottom row to the right, respectively number 1 and number 2. The neurons of the second row are number 3 and number 4 from left to right. Long hexagons of different colors represent the distance between two neurons. The darker the color, the greater the distance between the two neurons, that is, the greater the difference. We can judge the group with the biggest difference based on the color. In the sample clustering results of 2011, there is a significant difference in distance between No.1 and No.4 neurons, so two of the neurons are the low socioeconomic status group and the high socioeconomic status group (Fig. 1a). In the 2013 samples, No. 1 and No. 4 neurons have the largest distance and are regarded as the high socioeconomic status group and the low socioeconomic status group (Fig. 1b). In the 2015 samples, the distance between No.2 and No.4 neurons is black, so the samples in these two neurons have the largest difference (Fig. 1c). They represent the low socioeconomic status group and the high socioeconomic status group respectively. In the 2018 samples, as with the clustering results in 2011 and 2013, the distance between No.1 and No.4 neurons is the largest, which is regarded as the low socioeconomic status group and the high socioeconomic status group respectively (Fig. 1d).

## Results

### Descriptive statistics

Table 1 presents the descriptive statistics for each of the four waves of the CHARLS. These variables are used to analyze the concentration index and decomposition. In terms of basic situation, the average age of respondents is about 60. In terms of the economy, the average income and average expenditure of the respondents continue to increase. The average income increased from 7,891 to 17,852 as the average expenditure increased from 5,499 to 13,862. In terms of physical health, the annual change in self-reported health was not significant, and it was basically stable at around 3.1. However, the number of chronic diseases surged from 1.76 in 2015 to 3.33 in 2018. It may be related to the fact that most of the tracking samples entered the elderly after 7 years. And after entering old age, people will start to do physical examinations consciously, only to find that they have a certain chronic disease. In terms of medical insurance, with the implementation of the universal health care policy, the number of Chinese residents with medical insurance steadily increased. The average increased from 0.97 in 2011 to 1.03 in 2018. In terms of utilization of healthcare resources, the intra-group differences in outpatient care remained at a relatively stable level from 2011 to 2018, which the standard deviation for the utilization of outpatient care ranged from 0.4 to 0.44. The inpatient utilization rate gradually increases over time, which the average increased from 0.087 in 2011 to 0.19 in 2018. This may be related to the increase of age. The use of preventive healthcare does not change much from 2011 to 2018, and the differences (SD: 0.49 or 0.5) within the group remain at a relatively stable level, indicating that utilization of preventive care cannot be changed in a short period. It is related to people’s basic cognition.

**Table 1:** Sample descriptive statistics

Variables	2011 Mean(SD)/%	2013 Mean(SD)/%	2015 Mean(SD)/%	2018 Mean(SD)/%
**Demographic characteristics**				
Gender				
0 = male; 1 = female	0.48(0.50)	0.54(0.50)	0.53(0.50)	0.54(0.50)
Age				
Discrete variable	60.21(9.24)	59.74(9.54)	59.02(9.57)	60.42(9.45)
Education				
Illiterate (1 = Illiterate); Middle school and lower (2 = Did not finish primary, 3 = Sishu/home school, 4 = Elementary school, 5 = Middle school); High school and above (6 = High school, 7 = Vocational school, 8 = Two-/Three-Year Collage/Associate degree, 9 = Four-Year Collage/Bachelor’s degree, 10 = Master’s degree, 11 = Doctoral degree/Ph.D)	27.93;62.49;9.58	22.38;63.63;13.99	20.88;67.9;11.22	20.08;67.55;12.37
Residence location				
Urban zone (1 = Main city zone); Combination zone (2 = Combination zone between urban and rural areas, 3 = The town center, 4 = Zhenxiang area, 5 = Special area, 6 = Township Central); Rural zone (7 = Village)	8.56;24.98;66.46	15.76;26.53;57.71	9.13;27.64;63.23	14.04;27.2;58.76
**Economic level**				
Total income (year)				
Continuous variable	7891(12,758)	12,351(18,641)	12,346(27,901)	17,852(27,008)
Employment status				
Unemployment (0 = Unemployment, 3 = Retirement); Employment (1 = Informal employment, 2 = Formal employment, 4 = Re-employment after retirement)	3.96;96.04	18.63;81.37	7.82;92.18	16.32;83.68
Household facilities level				
Discrete variable	2.20(1.54)	2.97(1.52)	1.96(1.30)	3.37(1.32)
Total expenditure(year)				
Continuous variable	5499(9201)	9675(13,089)	11,051(23,133)	13,862(24,734)
**Health status**				
Number of chronic diseases				
Discrete variable	0.70(1.40)	1.65(1.60)	1.76(1.60)	3.33(2.64)
Self-reported health				
1 = Very good; 2 = Good; 3 = Fair; 4 = Poor; 5 = Very poor	3.19(0.82)	3.12(0.86)	3.15(0.90)	3.12(0.94)
ADL				
1 = With difficulty; 0 = Without difficulty	0.22(0.42)	0.21(0.40)	0.24(0.43)	0.21(0.41)
IADL				
1 = Difficult; 0 = Easy	0.22(0.42)	0.25(0.43)	0.28(0.45)	0.30(0.45)
**Insurance**				
Healthcare insurance number				
Discrete variable	0.97(0.29)	1.01(0.27)	0.97(0.57)	1.03(0.28)
**Healthcare service utilization**				
Outpatient level				
0 = No; 1 = Yes	0.26(0.44)	0.27(0.44)	0.23(0.42)	0.20(0.40)
Inpatient Level				
0 = No; 1 = Yes	0.087(0.28)	0.15(0.35)	0.14(0.35)	0.19(0.39)
Prevention				
0 = Didn’t take physical examination; 1 = Take a physical examination	0.46(0.50)	0.42(0.49)	0.39(0.49)	0.51(0.50)

### Homogeneous population recognition

**Fig. 2:**
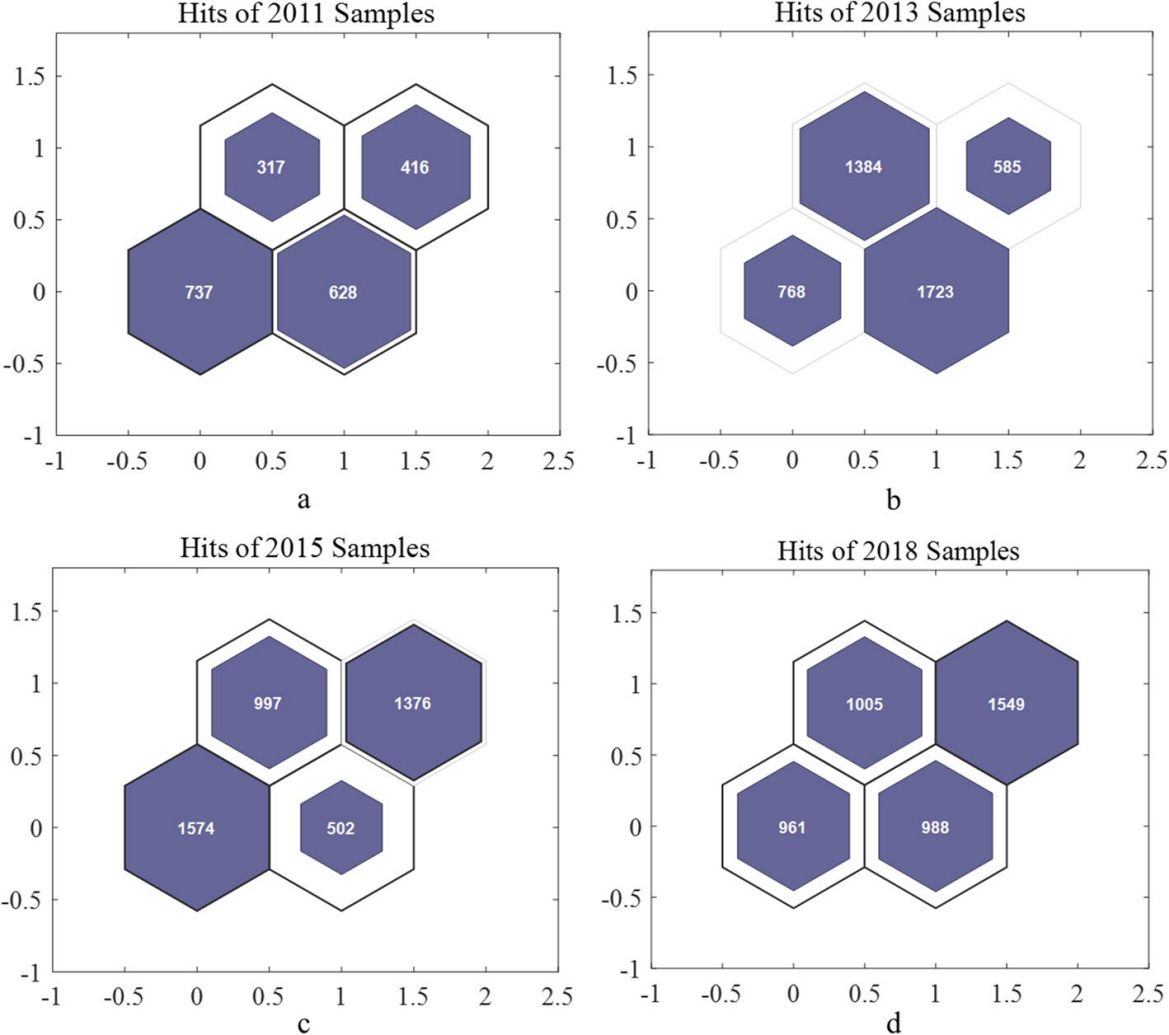
Hits of samples of four waves

**Fig. 3:**
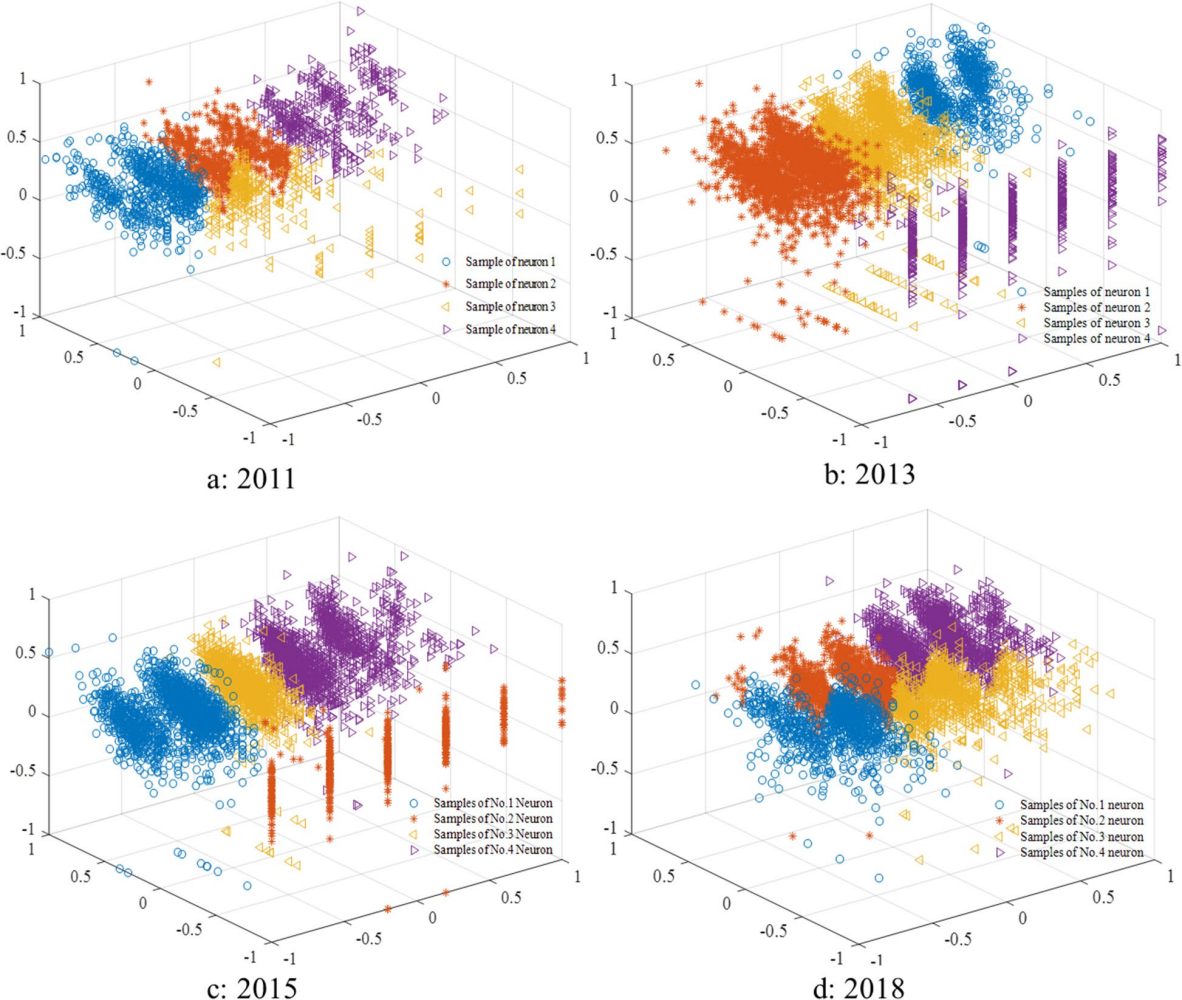
Samples distribution of four waves

The self-organizing maps and respondents’ distribution are shown in Figs. 2 and 3. In 2011, the respondents of No.1 neuron and No.4 neuron were the low socioeconomic status group and the high socioeconomic status group respectively, and the corresponding respondents sizes were 737 and 416 (Figs. 2a, 3a); In 2013, the respondents on No.1 neuron’ respondents size is 768. The respondents on No.4 neuron were regarded as the low socioeconomic status group, with respondents size of 585 (Figs. 2b, 3b). In 2015, the distance between No.2 neuron and No. 3 neuron was the largest, so these respondents are divided into the low socioeconomic status group and the high socioeconomic status group, which the number is 502 and 1376 (Figs. 2c, 3c). In 2018, it is similar to 2011 and 2013 situation. The No.1 and No.4 neuron is recognized the low socioeconomic status group and the high socioeconomic status group, and the sample sizes are 961 and 1549 respectively (Figs. 2d, 3d).

## Annual changes in inequality of samples

**Fig. 4:**
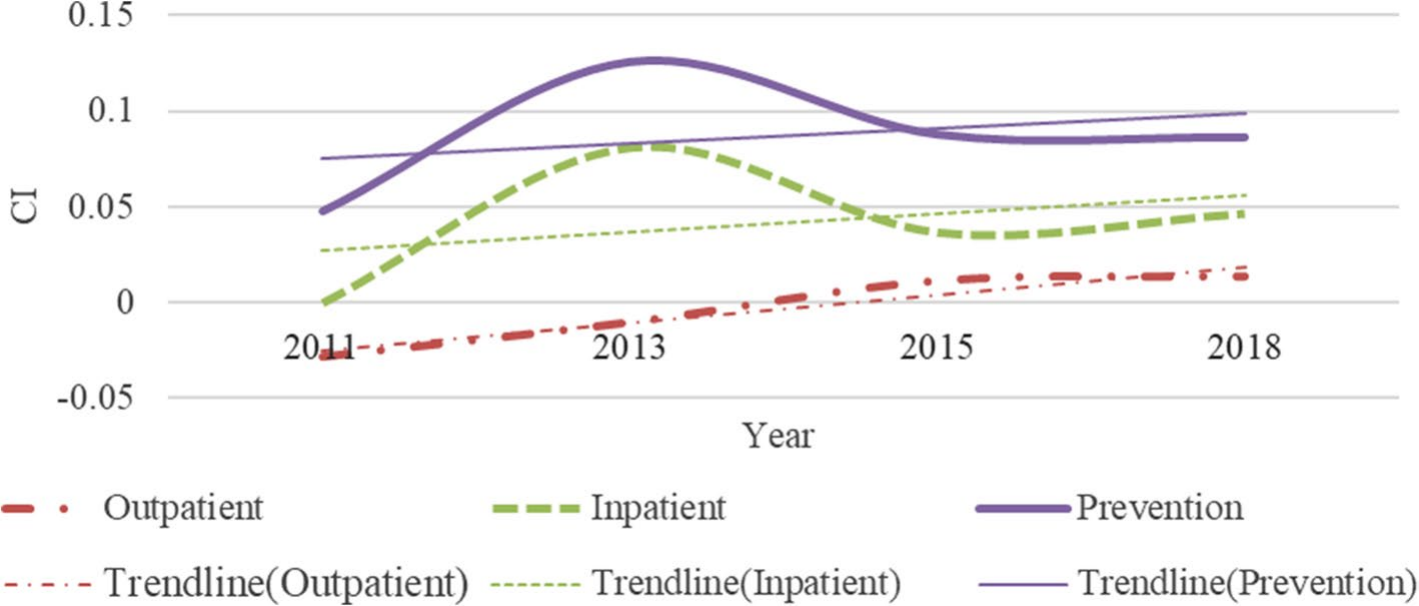
Annual change in inequality

Our results show significant inequality inclined to high socioeconomic status in the use of inpatient and preventive care in middle-aged and older adults in China (Fig. 4). And the situation is the same every year. For outpatient care, the CI changed from -0.0281 in 2011 to 0.0136 in 2018 (Table 2), which indicates a change from pro-poor inequality to pro-rich inequality. Yuan’s research also shows that the utilization of outpatient care in China in 2011 showed pro-poor inequality [47]. CI for inpatient and preventive care are all positive and statistically significant except for the inpatient care’s CI in 2011, indicating richer individuals use disproportionately more health services than others. Xu and Fu’s research also shows that there are pro-rich inequalities between these two types of healthcare [48, 49]. While lower socioeconomic status groups access a significantly lower share of healthcare resources, irrespective of service type. In contrast, outpatient cares in 2011 and 2013 were disproportionately inclined to the poor (CI: -0.0281 in 2011; -0.0102 in 2013). And outpatient care shows pro-rich inequality since 2015. This finding is consistent with Fu’s research [50]. In the three types of healthcare, preventive care has the highest degree of inequality, followed by inpatient, and outpatient care tends to pay more attention to equality. This shows that outpatient and inpatient care, as basic healthcare services, and the Chinese government has controlled the inequalities to a certain level through medical insurance policies.

**Table 2:** Inequality in healthcare utilization

**Outpatient**	**Concentration Index**	**Std.Err**	***p*-value**
2011	-0.0281	0.0054	0.0000
2013	-0.0102	0.0035	0.0032
2015	0.0114	0.0038	0.0031
2018	0.0136	0.0040	0.0006
**Inpatient**	**Concentration Index**	**Std.Err**	***p*-value**
2011	0.0058	0.0082	0.4784
2013	0.0812	0.0048	0.0000
2015	0.0369	0.0055	0.0000
2018	0.0468	0.0059	0.0000
**Prevention**	**Concentration Index**	**Std.Err**	***p*-value**
2011	0.0479	0.0024	0.0000
2013	0.1255	0.0027	0.0000
2015	0.0878	0.0028	0.0000
2018	0.0863	0.0024	0.0000

From the perspective of time, the inequality trend in outpatient care was found to have the most changes from -0.0281 to 0.0136. However, compared with 2011, the degree of inequality in outpatient care eased in 2013 (CI: -0.0281 ~ -0.0102), 2015 (CI: -0.0281 ~ 0.0114), and 2018 (CI:-0.0281 ~ 0.0136). The trend of gradual reduction in the inequality of outpatient care is also seen in Fan’s findings from 2011 to 2018 [51]. This shows that during the period from 2011 to 2018, the Chinese government’s medical policies had a more obvious effect when trying to ease the inequality of outpatient care utilization. In 2013, the inequality degree of inpatient and preventive care was the highest, and in 2015 and 2018, the degree of inequality decreased. This shows that relevant national policies had better results in 2015 and 2018. But beware of re-increasing inequality in hospitalization. Since the data is obtained through longitudinal survey, there is no excessive difference in individual consciousness caused by different samples that leads to unreliable results. It is more accurate to estimate the degree of inequality in use of inpatient and preventive care.

## Decomposition of inequalities in healthcare utilization

**Fig. 5:**
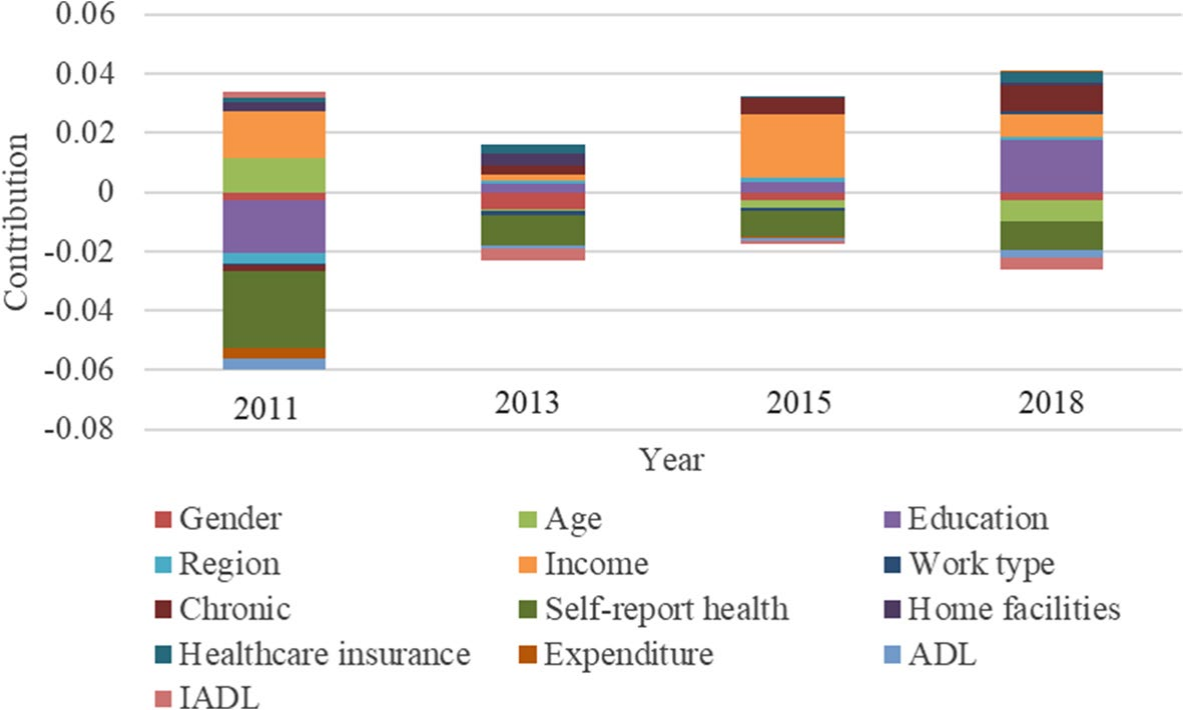
Decomposition of the CI in outpatient care

**Fig. 6:**
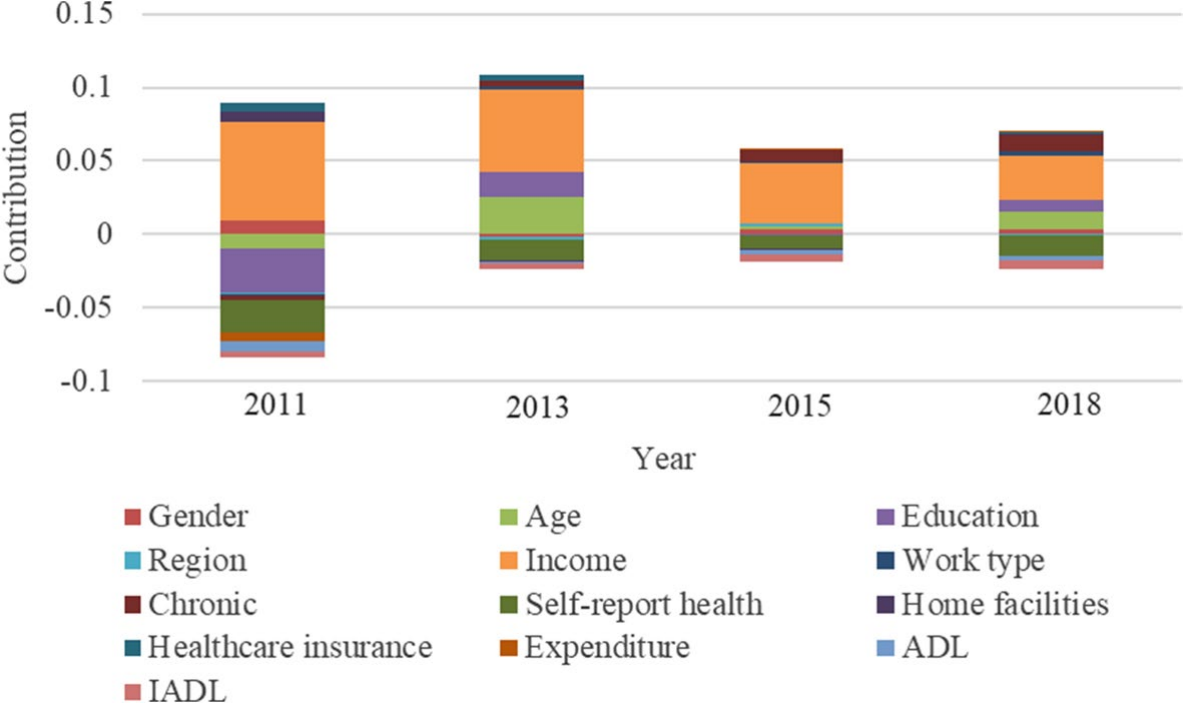
Decomposition of the CI in inpatient care

**Fig. 7:**
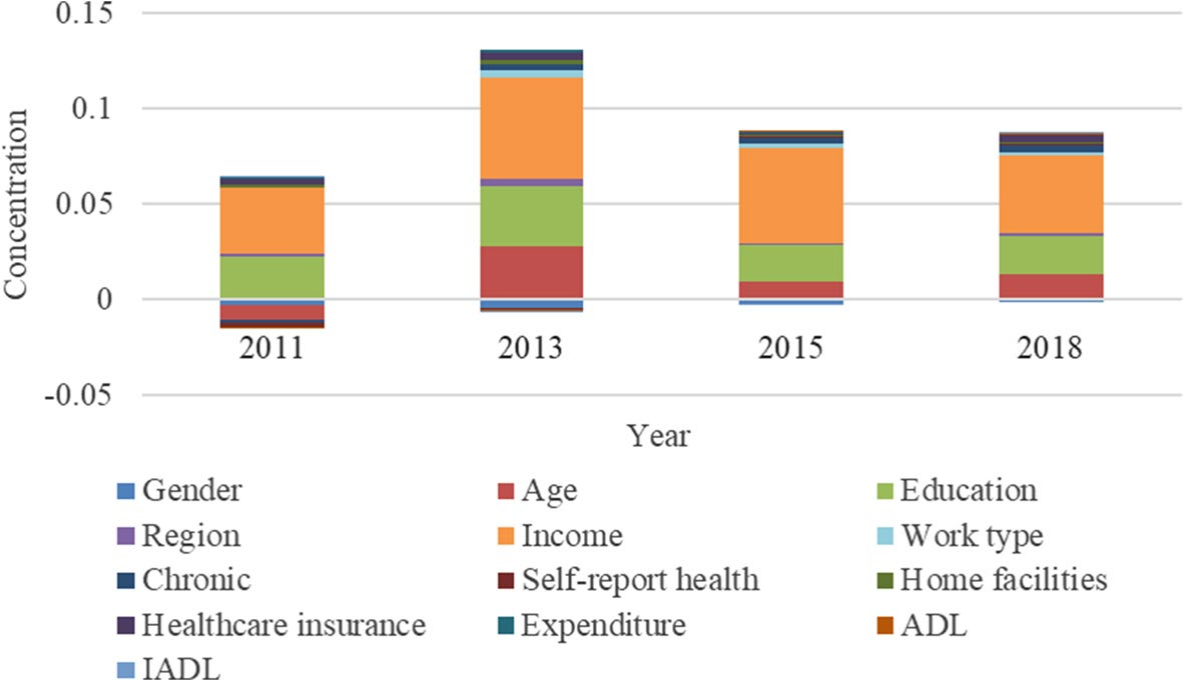
Decomposition of the CI in preventive care

Figures 5, 6 and 7 present the decomposition of the CI into the contributions of four types factors. It specifically includes the basic characteristics of the population, socioeconomic conditions, insurance and medical needs (physical status). The three figures respectively represent the contribution of outpatient, inpatient, and preventive care. The distribution of the columns from 0 tick mark indicate that different contribution of factors can pull inequality either towards richer individuals (positive values, above-hand side) or poorer individuals (negative values, below-hand side). The height of the column representing each factor indicates its contribution to the total CI, that is, a tall column means that its contribution is higher than other factors.

The magnitude of the contribution of each factor depends on: (i) how sensitive health care use is to variation in the given factor (i.e., its elasticity with respect to it); and (ii) how equal the distribution of a given factor is with respect to the socioeconomic status of a household (i.e., its CI). Consequently, the largest contributions to overall inequality are relative to those factors that are both unequally distributed and strongly associated with healthcare utilization.

In Fig. 5, the main driving factors of socioeconomic inequality in outpatient care in China are age, education, income, chronic diseases and self-reported health. The education and age of the individuals using health services are particularly relevant for the distribution of outpatient care utilization, whereas individuals’ characteristics explain a large portion of inequality in outpatient care utilization in 2011 and 2018. Liu and Guo’s research results confirm that education and age have a greater impact on the inequality in outpatient care utilization [52, 53]. In this study, as it should be noted that in 2011, the contribution of age was positive (0.0114), and the contribution of education was negative (-0.0179). However, in 2013, 2015 and 2018, the contributions of age and education were opposite to the 2011 performance. The contribution of age in the three waves’ surveys were -0.0004, -0.0025, -0.0069. The contributions of education in the three waves’ surveys were 0.0028, 0.0036 and 0.0174. This means that with age, it helps to alleviate socioeconomic inequality in outpatient care utilization. Higher education levels increase inequality. This shows that for people who are gradually entering the elderly group, the age difference has little effect on the inequality of outpatient care. The main factor that motivates them to frequently use outpatient care is their medical awareness, that is, the potential impact of education on the medical awareness of middle-aged and elderly people. Income is an important factor in the measurement of economic level. No matter what year, it will increase the inequality of outpatient care utilization. This result is confirmed by empirical studies in different countries [53, 54]. Chronic diseases and self-reported health are variables in measuring health status, that is, medical needs. In particular, chronic diseases gradually aggravated the inequality of outpatient care utilization over time (2013 ~ 2018: 0.0032 ~ 0.0086). This shows that chronic diseases of the elderly have a greater impact on their normal life with increasing age, causing them to use outpatient care more than those without chronic diseases. The contribution of self-reported health in the four waves is negative (2011 ~ 2018: -0.0261 ~ -0.0097), suggesting that the larger the self-reported health value is, the more conducive it is to alleviate the inequality of outpatient care utilization. This suggests that it does not exacerbate inequality in outpatient utilization among population with poor physical health. As a basic medical service, outpatient care requires minimum payment for patients, attracting most people with poor self-reported, which helps alleviate inequality in outpatient utilization.

In Fig. 6, for inpatient care, the main driving factors of socioeconomic inequality are age, education, income, chronic diseases, and self-reported health. These factors explain a large portion of inequality in inpatient care utilization. The contribution of age in 2013, 2015, and 2018 were 0.0253, 0.0027 and 0.0117, which means that older age exacerbated the inequality of inpatient care. This is the opposite of its impact on outpatient utilization. The contribution of education to the inequality of inpatient care varies in the four waves, -0.0296, -0.0008 in 2011 and 2013, 0.0172, and 0.0083 in 2013 and 2018, respectively. While Luo’s research shows that people with less education have lower rates of inpatient utilization [55]. This is similar to the 2013 and 2018 results in this study. But education is also usually correlated with income [56], so it is possible to have a negative contribution. The contribution of income to the inequality of inpatient care is positive in four waves. But its value keeps declining, with contributions ranging from 0.0679, 0.0556, 0.0409, and 0.0294 in four waves of surveys. This shows that the extent which income inequality contributes to the inequality of inpatient care is decreasing with increasing age. This, on the other hand, reflects the nature of necessities for inpatient care with increasing age. On the whole, compared with inequality of outpatient care, income contributes more to the inequality of inpatient care than that in outpatient care. The contribution of chronic diseases was positive in 2013 (0.0040), 2015 (0.0077), and 2018 (0.0113), which means that the difference in chronic diseases exacerbated the inequality of inpatient care inclined to high socioeconomic status. This result was also confirmed by Guo’s research [53]. And as time changes, the contribution rate of chronic diseases continues to increase. This shows that with the increase of age, middle-aged and older adults with more chronic diseases use more inpatient care, compared with those without chronic diseases.

In Fig. 7, the main driving factors of socioeconomic inequality in preventive care utilization in China are also age, education and income. These three factors explain the large proportion of inequality inclined to high socioeconomic status. The contribution of income is the most significant, and the contributions in the four waves were 0.0346, 0.0525, 0.0496, and 0.0408 respectively. Next is education and age. The contribution of income and education to inequality in preventive care utilization was also demonstrated in empirical research in Kenya [13]. The contribution of age to the inequality in preventive care is similar to that of inpatient care. The contribution of education is positive in the four-wave surveys, which are 0.0221, 0.0313, 0.0189, and 0.0201. This shows that education could exacerbate inequality in preventive care utilization. In particular, compared with outpatient and inpatient care, education contributes more to the preventive care utilization inequality.

## Inequality among the homogeneous population

### High socioeconomic status group

**Fig. 8:**
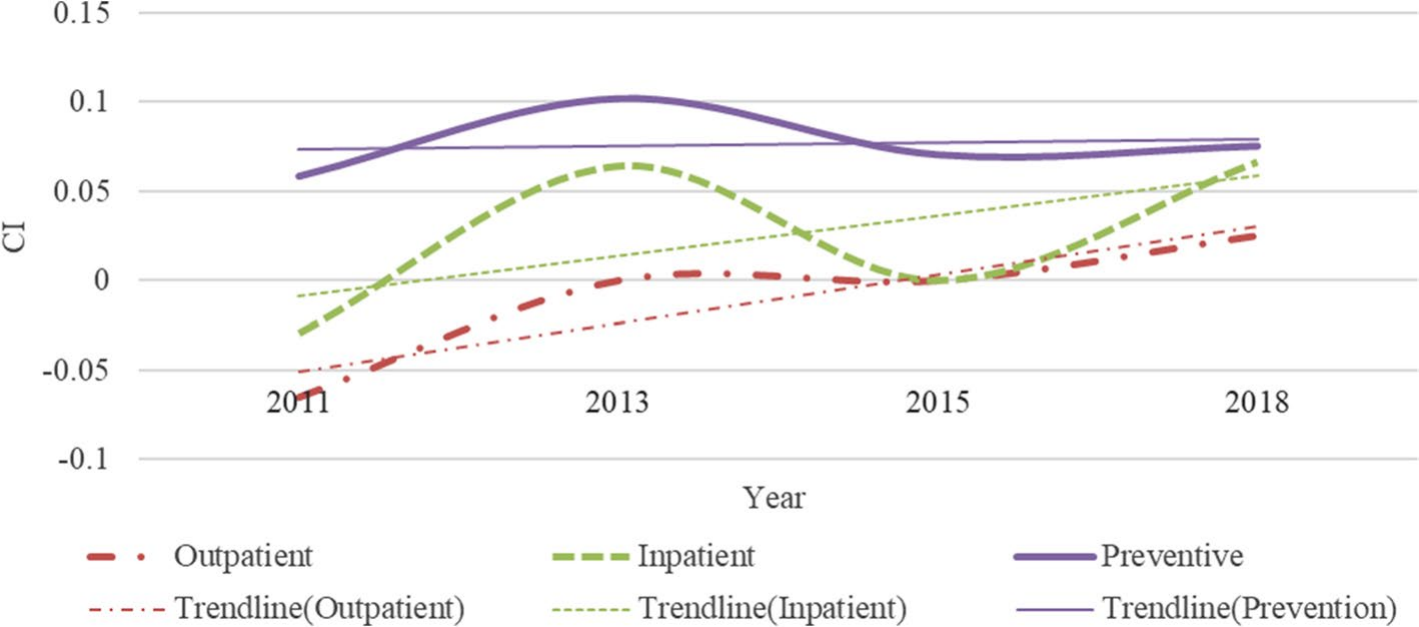
Annual change in inequality among high socioeconomic status groups

In the high socioeconomic status group shown in Fig. 8, the inequality trend of all types of medical resources is similar to the full samples. Outpatient utilization is the most equal, inpatient care is moderately unequal, and the most unequal is preventive care. In more detail, there are three differences in the inequality of the high socioeconomic status group compared with the results of the full samples. Firstly, the outpatient utilization of the high socioeconomic status group transitioned from being pro-poor to being equal in 2013 (CI: -0.0654 ~ 0.0000). In the full samples, there is still a slight inequality inclined to the poor in 2013 (CI: -0.0281 ~ -0.0102). Secondly, in 2011, the utilization of inpatient care in the high socioeconomic status group showed pro-poor inequality (CI: -0.0296). There was no significant inequality in inpatient care of the full samples. Thirdly, for preventive care, the annual trend of inequality in the high socioeconomic status group is relatively flat, while the annual change of the full samples is larger.

### Low socioeconomic status group

**Fig. 9:**
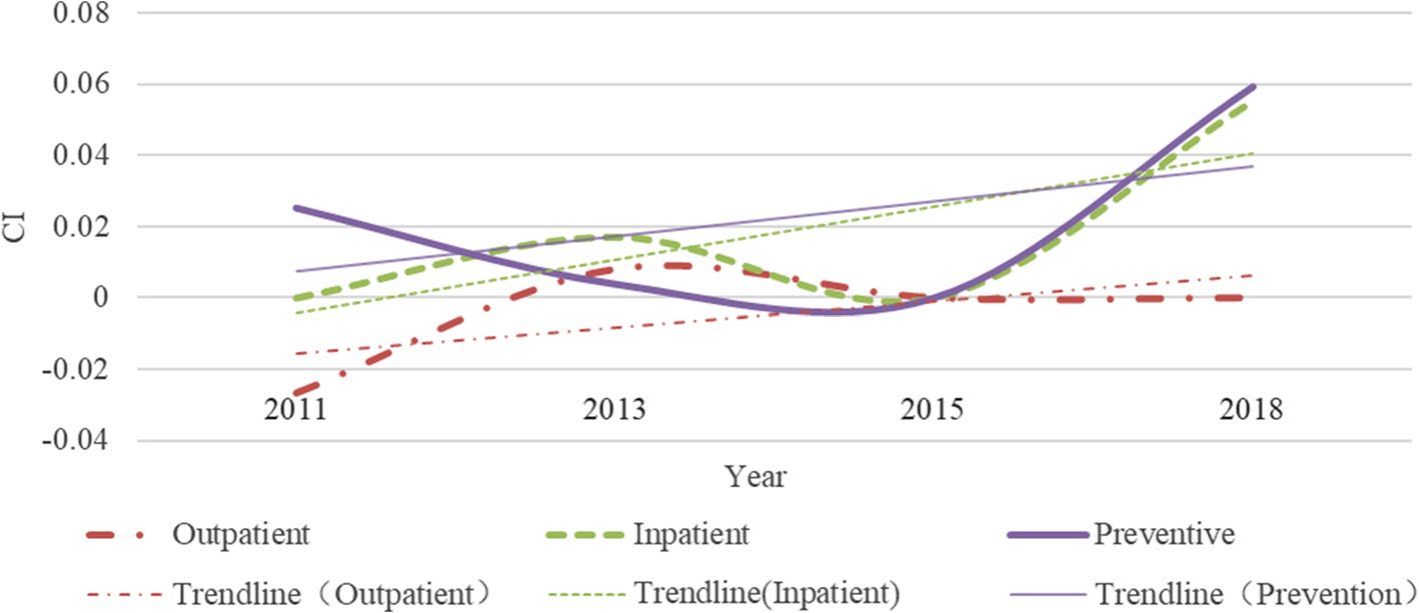
Annual change in inequality among low socioeconomic status groups

Inpatient care and preventive care resources are more inclined to the rich people in the low socioeconomic status group. The utilization of outpatient care is slightly inclined to the poor. According to the trend line in Fig. 9, the inequality degree of utilization in preventive care is roughly the highest (CI: 0.0252 ~ 0.0592 from 2011 to 2018), followed by inpatient care (CI: 0.0000 ~ 0.0554 from 2011 to 2018), and outpatient care is the most equal (CI: -0.0267 ~ 0.0000 from 2011 to 2018). The development of inequality between preventive and inpatient care is on the rise. Among them, preventive care reached its peak in 2018. However, outpatient care became equal year by year (CI: -0.0267 ~ 0 from 2011 to 2018).

### Comparative analysis

For the three types of healthcare utilization, the inequality degree of the high socioeconomic status group is higher than that of the low socioeconomic status group approximately. Inconsistent with the performance of the high socioeconomic status group is that the inequality of preventive care in the low socioeconomic status group is lower than that of outpatient and inpatient care in 2013 (CI: Inpatient: 0.0171 < Outpatient: 0.0081 < Prevention: 0.0039), while the annual inequality of preventive care in the high socioeconomic status group was greater than that of outpatient and inpatient care. In 2015, there was no obvious inequality in the three types of medical care in the low socioeconomic status group. In the high socioeconomic status group, only outpatient and inpatient care are not significantly unequal. It can be seen that the inequality degree of preventive care utilization is greatly affected by policy changes in the low socioeconomic status group. In the high socioeconomic status group, the richer the higher the inequality degree in preventive care (CI: 0.0588, 0.1020, 0.0707 and 0.0755, respectively in four waves). This shows that when the economic level reaches a certain level, the impact of preventive care at the economic level will increase significantly. Secondly, the utilization of outpatient care in the high socioeconomic status group shows inequality inclined to high socioeconomic in 2018 (CI: 0.0253), while the utilization of outpatient care in the low socioeconomic status group tended to be equal year by year since 2015 (CI: 0.0000 ~ 0.0000 from 2015 to 2018). This result manifests that the high socioeconomic status group has greater economic freedom, so the difference in the utilization of outpatient care varies greatly with the economic level. On the contrary, in the low socioeconomic status group, the degree of economic freedom is less, and it is a good state to be able to meet the use of basic medical services.

## Discussion

In this study, we estimated the changes in the inequality degree in outpatient, inpatient, and preventive care use among middle-aged and elderly people in China from 2011 to 2018, and further estimated the changes in inequality among homogeneous population. We also explored the contribution of related factors to the inequality degree. Our study has three important findings.

First, in the measurement of the full samples’ inequality, we observed that preventive care has the largest pro-rich inequality and outpatient care has the smallest inequality. Preventive care utilization, as improved medical service, is sensitive to people’s socioeconomic level, so it has the highest inequality degree. However, the inequality appeared to be eased in 2013, which may be related to the family doctor contract services (FDCS) launched in 2009 in China, which the pilot services started in 2012. This service was designed to focus on the utilization of the community medical service [57], including simple and free physical examinations for some chronic diseases. People with chronic diseases are more motivated to participate in this service [58]. With the popularization of FDCS, the number of people covered increased gradually, especially among middle-aged and elderly people with chronic diseases. And according to our findings, the average number of chronic diseases in the sample increased over year. This led to the degree of inequality in preventive care showed a significant decline in 2015. The FDCS was officially implemented nationwide since in 2016 [59]. In addition, China introduced another policy of free medical examinations for middle-aged and elderly people in 2017, which also promoted the utilization of preventative care to alleviate the impact of economic levels on their utilization. Thus, the degree of preventive care inequality in 2018 was slightly lower than that in 2015. The overall inequality of outpatient care is relatively low, and it tends to become inequality inclined to high socioeconomic status. The low threshold fee for outpatient visits is an important reason for the low level of inequality. From 2011 to 2018, the inequality changed from inclining to low socioeconomic status to inclining to low socioeconomic status. This shows that the utilization of outpatient care in China is gradually restricted by economic levels. Compared with outpatient, inpatient care has a higher degree of inequality. The generally high cost of inpatient care is an important factor causing the pro-rich inequality. And the annual change trend of inpatient care is similar to that of preventive care. This shows that in addition to the supplement of medical insurance to the use of inpatient, preventive care may be a key factor in avoiding the utilization of inpatient services.

Second, by decomposing the concentration index, we found that compared with preventive care, self-reported health has a greater contribution to the inequalities of outpatient and inpatient care. This once again reflects the basic and necessary characteristics of outpatient and inpatient care. The utilization is closely related to personal health. It should be noted that compared with inpatient care, self-reported health has a higher contribution to the utilization of outpatient care, and it is the highest contribution rate of all influencing factors in outpatient care. This further proves that the personal health status is the first driving force for outpatient utilization. For inpatient care, income is the highest contribution rate of all influencing factors, which is higher than that of self-reported health. This explains that inpatient care is more restricted by economic conditions. This is the same as the previous study of inpatient care [60]. And it is consistent with the conclusion that the degree of inequality in inpatient care is higher than that in outpatient care in the previous analysis. Income is also the highest contribution rate among all factors of preventive care. Different from outpatient and inpatient care is that another major factor affecting preventive care is education. Personal health status is not a key factor in determining whether middle-aged and older adults use preventive care. Their health awareness and preventive awareness have become more important. And the main factor affecting health awareness and preventive awareness is education level, so the unequal utilization of medical resources caused by differences in education level is more prominent in preventive care. Another factor that needs attention is age. The contribution of age to the inequality of the three types of medical resource utilization is smaller than the previous two factors, but it is more obvious on the whole. The age difference brings about the difference in physical function and the difference in health awareness given by the times. In particular, chronic diseases made a greater contribution to the inequality of utilization in outpatient and inpatient care in 2015 and 2018. There may be two reasons for this situation: one is that as the age of the respondents increases, the chronic disease appears; the other is that as the Chinese government gradually popularizes the management of chronic diseases for middle-aged and elderly people, people are beginning to actively treat and prevent chronic diseases. So the presence or absence of chronic diseases will affect the unequal utilization of medical resources.

Third, among the homogeneous population, we observed that the inequality in the medical resources utilization of the high socioeconomic status group is higher than that of the low socioeconomic status group. This explains why a high socioeconomic level could exacerbate the utilization inequalities among homogeneous population. From the analysis of the inequality contribution, it can be seen that the contribution of income to the unequal utilization of the three types of medical resources is more obvious. This suggests that the higher the income is, the higher the degree of inequality it will cause. Adjusting subsidies and utilization methods according to different economic levels can help effectively alleviate inequality. We also found that in the low socioeconomic status group, the utilization of outpatient care is slightly inclined to the poor and tends to be equal over time. Other types of healthcare have a disproportionate tend to the rich, and the degree of inequality is slightly higher. In the high socioeconomic status group, outpatient and inpatient care transitioned from pro-poor inequality to pro-rich inequality from 2011 to 2018. When an individual’s economic conditions reach a certain level, its inequality impact exceeded the control of the policy. Its performance is especially significant in outpatient care utilization. And the inequality degree of outpatient care is the lowest in the rich and low socioeconomic status group, indicating that the current equalization and accessibility of outpatient care are relatively good. Yan’s research also points to an increase in outpatient visits as medical insurance coverage improves, suggesting improved accessibility [61]. The inequality degree of inpatient care in 2018 was higher in both the low socioeconomic status group and the high socioeconomic status group. It is necessary to guard against the increase in inequality of inpatient care in recent years. This shows that the current medical policy cannot balance the inequality of inpatient care, regardless of which homogeneous group. And other scholars also proved that the poor in China are always more likely to forgo inpatient care than the rich [61], which may result in socioeconomic inequality. It is worth noting that in 2015, there was no obvious inequality in preventive care in the low socioeconomic status group, while the inequality in the high socioeconomic status group was high. Combined with the introduction of the free medical examination policy that was analyzed in the previous study, we find that the inequality of the low socioeconomic status group is more elastically affected by the welfare policy, and the high socioeconomic status group is less elastic.

Our study has important documentary value for understanding the differences in the inequality degree among the homogeneous population. At present, most of the literature measuring the inequality of healthcare utilization focuses on the estimation of all groups, ignoring the existence of inequality among homogeneous groups. But this is of great significance for the formulation of medical and related policies, because the inequality in the use of healthcare caused by the huge income gap cannot be solved by a single medical policy. The government can only guarantee relative equality in medical treatment. In addition, SOM is introduced into the research framework to enrich the research on concentration index.

At the same time, this study also has reference value for other countries in the world, especially those that are unable to achieve high-quality UHC or those with insufficient health spending. Temporarily setting the near-term goal of achieving relative inequality among homogeneous population is an effective way to alleviate inequalities. Because income inequality caused by economic development in many other countries leads to unequal utilization of medical resources, such as Iceland and Ireland [11], Kenya [13], Brazilian [17], etc. However, there are differences in the inequality of healthcare utilization affected by income in developed and developing countries. Developed countries are more affected by healthcare spending policy [11], and the inequalities created by a temporary economic crisis can be eliminated. And developing countries are still working towards UHC [31]. Government spending on health cannot reach a better level in a short period, and income inequality brought about by economic development cannot be alleviated in a short period. When determining inequality between a homogeneous group and full samples, on the one hand it becomes clear how the limited health expenditure can be used to achieve the greatest benefit, and on the other hand, it becomes clear which medical service needs more attention. Therefore, this study may be more meaningful for developing countries.

However, some limitations of this study should be acknowledged. First, the estimate of preventive care utilization is relatively loose. This paper uses the interval of each follow-up survey as the measurement standard, that is, this indicator is that preventive care was used during the interval (2–3 years), so the estimated use rate is relatively high. Second, homogeneous population identification only recognizes people with similar economic levels, and the utilization of healthcare is also greatly affected by other conditions. However, these limitations do not invalidate our work, and the nature of large samples reduces estimation bias to some extent, as does the use of panel data.

Future research could identify homogeneous population in terms of physical condition, medical insurance, etc., to further examine healthcare utilization inequalities. The selection of dimensions can be determined according to the most critical factors affecting the inequality of healthcare utilization in the region. Another possible direction is multi-dimensional homogeneous population identification, examining the utilization of healthcare in similar population across multiple dimensions. This addresses situations where a single dimension is not comprehensive or where multiple dimensions are equally important to impact on healthcare inequalities.

## Conclusions

Although in 2009, NHCR made breakthroughs in healthcare, medicine, and health insurance, such as promoting universal medical insurance, improving health insurance levels, promoting the hierarchical medical system, and strengthening medical service capabilities, etc. [62]. These unified reform measures can be applied regardless of their socioeconomic status. However, the problem of expensive medical care in China still exists [63], that is, the unequal utilization of healthcare services caused by differences in economic levels. Because the difference in socioeconomic is difficult to achieve a complete balance, it is important to achieve relative equality, that is, to achieve relative equality between homogeneous groups of people based on basic medical and health services for everyone. By measuring the degree of inequality between the full samples and the homogeneous population, the contributory factors of inequality and the inequality difference among the homogeneous population are clarified. Accordingly, three policy implications could be put forward from our study:

(i) Further promote free medical examination services for the elderly. Through the establishment of a physical examination card, a free physical examination service is issued on the card once a year, to urge the elderly to use preventive medical treatment, improve the awareness of health prevention for the elderly, and combine prevention and treatment to improve the overall health level. This has a positive impact not only on alleviating inequality in preventive care utilization due to insufficient income among the low socioeconomic group. It is also possible to urge group with high socioeconomic status to use basic free preventive services to alleviate the large inequality caused by differences in health awareness. In particular, the promotion of high-quality free preventive care is more attractive to groups with high socioeconomic status. Health departments can optimize free preventive services, such as screening for common chronic diseases and routine monitoring, based on the needs of most older adults.
(ii) Advocate various forms of CCB activities (Collaboration for Community Benefits) [64], which can improve the overall health of the community through health lectures, community construction activities, donations of first aid facilities, etc. To correct people’s excessive utilization in the high socioeconomic status group or insufficient utilization in the low socioeconomic status group. Appropriate healthcare utilization not only has a positive effect on resource optimization, but also helps to eliminate the utilization inequalities due to health awareness among homogeneous population.
(iii) The government could control the difference in terms of reimbursement and out-of-pocket, by combining the improvement of the medical insurance reimbursement regulations and the control of the cost of drugs and inspections [65]. Among them, in medical reimbursement, free medical insurance should be provided for those who cannot afford to pay, and a cheap medical system should be established through the identification of the medical insurance card. This may be more helpful in alleviating utilization inequalities among the low socioeconomic status group.

## Data Availability

The datasets generated and analysed during the current study are available in the CHARLS repository, [http://charls.pku.edu.cn/en].

## Abbreviations

CI: Concentration Index
CCB: Collaboration for Community Benefits
NHCR: New Health Care Reform
COVID-19: Corona Virus Disease 2019
CHARLS: China Health and Retirement Longitudinal Study
UHC: Universal Healthcare
SOM: Self-organizing Maps
FDCS: Family Doctor Contract Services

## References

[1] World Health Organization (1978). Declaration of Almata.

[2] Quigley J, Morsink J (1999). The universal declaration of human rights: origins, drafting, and intent. Am J Leg Hist.

[3] Cheng FZ (2006). On the reasons and countermeasures for the difficulty and expensive medical treatment of the masses. China Hospital.

[4] Wang BZ (2007). Analysis of the health economy of "seeing a doctor is expensive and difficult". Chinese Health Economics.

[5] Zhu HP (2017). Strengthening and innovating social governance and improving the medical insurance system for urban and rural residents-learning experience from the report of the 19th National Congress of the Communist Party of China. Econ Perspect.

[6] Cernadas A, Fernandez A (2020). Healthcare inequities and barriers to access for homeless individuals: a qualitative study in Barcelona (Spain). Int J Equity Health.

[7] Liu D. The status quo and trends of China’s population aging. China Net. 2020. Available at http://www.cctv.com/special/1017/-1/86774.html. Accessed 13 Apr 2022.

[8] Liu S, Coyte PC, Fu MQ (2021). Measurement and determinants of catastrophic health expenditure among the elderly households in China using longitudinal data from the CHARLS. Int J Equity Health.

[9] National Health Commission Statistical Information Center. China Health Statistics Yearbook. Beijing: Peking Union Medical University Press. 2019;2019.

[10] Fourie J, Jayes J (2021). Health inequality and the 1918 influenza in South Africa. World Dev.

[11] Torfs L, Adriaenssens S, Lagaert S (2021). The unequal effects of austerity measures between income-groups on the access to healthcare: a quasi-experimental approach. Int J Equity Health.

[12] Saito MK, Quaresma M, Fowler H (2021). Exploring socioeconomic differences in surgery and in time to elective surgery for colon cancer in England: population-based study. Cancer Epidemiol.

[13] Ilinca S, Giorgio LD, Salari P (2019). Socio-economic inequality and inequity in use of health care services in Kenya: evidence from the fourth Kenya household health expenditure and utilization survey. Int J Equity in Health.

[14] Cutler DM, Lleras-Muney A, Vogl T. Socioeconomic status and health: dimensions and mechanisms. Cambridge: National Bureau of Economic Research; 2008. p. 14333.

[15] Tian F, Pan J (2021). Hospital bed supply and inequality as determinants of maternal mortality in China between 2004 and 2016. Int J Equity Health.

[16] Bg E, Ws J, Pdm P (2021). Health-care guidelines and policies during the COVID-19 pandemic in Mexico: a case of health-inequalities. Health Policy Open.

[17] Galvao MHR, Roncalli AG (2021). Does the implementation of a national oral health policy reduce inequalities in oral health services utilization? The Brazilian experience. BMC Public Health.

[18] Rivillas JC, Deviarodriguez R, Ingabire MG (2020). Measuring socioeconomic and health financing inequality in maternal mortality in Colombia: a mixed methods approach. Int J Equity Health.

[19] Gong SZ, Gao Y, Zhang F (2021). Evaluating healthcare resource inequality in Beijing, China based on an improved spatial accessibility measurement. Trans GIS.

[20] Gu H (2019). Coordinating the urban and rural medical insurance system, income-related medical service utilization and health inequality. Social Sci J.

[21] Deng XJ, Wang XY (2020). Inequality measurement and decomposition of rural household sanitation facilities. Statistics and Decision.

[22] Gu MY. Dictionary of Education. Shanghai: Shanghai Education Press; 1998. p. 1998.

[23] Erreygers G (2013). A dual Atkinson measure of socioeconomic inequality of health. Health Econ.

[24] Stefan S, Dan O, Boris C (2021). Social protection expenditure on health in later life in 20 European countries: Spending more to reduce health inequalities. Soc Sci Med.

[25] Heaton TB, Crookston B, Pierce H (2016). Social inequality and children's health in Africa: a cross sectional study. Int J Equity Health.

[26] Young RA (2021). What do we mean, 'necessary'?-Achieving balance and recognizing limits in primary healthcare and universal healthcare. J Eval Clin Pract.

[27] Krech R, Kickbusch I, Franz C (2018). Banking for health: the role of financial sector actors in investing in global health. BMJ Glob Health.

[28] Zimbabwe National Health Financing Policy : Resourcing Pathway to Universal Health Coverage 2016 - Equity and Quality in Health : Leaving No One Behind (English). Washington, D.C. : World Bank Group. 2019;2019. http://documents.worldbank.org/curated/en/840661563174110288/Zimbabwe-National-Health-Financing-Policy-Resourcing-Pathway-to-Universal-Health-Coverage-2016-Equity-and-Quality-in-Health-Leaving-No-One-Behind

[29] Deloitte (2014). 2015 healthcare outlook.

[30] Narayana MR (2016). India's proposed universal health coverage policy: evidence for age structure transition effect and fiscal sustainability. Appl Health Econ Health Policy.

[31] Cotlear D, Nagpal S, Smith O (2015). How 24 developing countries are implementing universal health coverage reforms from the bottom up.

[32] Qi LS, Li ZN (2011). Income-related health and medical service utilization mobility. Econ Res.

[33] Van DE, Jones AM (2003). Inequalities in self- reported health: validation of a new approach to measurement. J Health Econ.

[34] Zhao GC, Gu H (2016). Health insurance and decomposition of the inequality in health care utilization. Zhejiang Soc Sci.

[35] Jenkins S (1988). Calculating income distribution indices from Microdata. Natl Tax J.

[36] Kakwani NC (1980). Income inequality and poverty: methods of estimation and policy applications.

[37] Lerman RI, Yitzhaki S (1989). Improving the accuracy of estimates of gini coefficients. J Econometrics.

[38] Dong YQ, Deng QH, Li SP (2022). The health inequality of children in China: a regression-based decomposition analysis. Child Indic Res.

[39] O'Donnell O, Doorslaer EV, Wagstaff A (2008). Analyzing health equity using household survey data. World Bank.

[40] Wagstaff A, van Doorslaer E, Watanabe N (2003). On decomposing the causes of health sector inequalities with an application to malnutrition inequalities in Vietnam. J Econometrics.

[41] Mayaud JR, Tran M, Nuttall R (2019). An urban data framework for assessing equity in cities: comparing accessibility to healthcare facilities in Cascadia. Comput Environ Urban Syst.

[42] Kohonen T (2001). Self-organizing maps.

[43] Johnsson M. Applications of self-organizing maps. London: InTech Open; 2012. p. 2012.

[44] Vaz E, Bacao F, Damasio B (2021). Machine learning for analysis of wealth in cities: a spatial-empirical examination of wealth in Toronto. Habitat Int.

[45] Mayaud JR, Anderson S, Tran M, Radic V (2019). Insights from self-organizing maps for predicting accessibility demand for healthcare infrastructure. Urban Science.

[46] Deng XJ, Wang XY (2020). Inequality measurement and decomposition of rural household environmental sanitation facilities. Statistics Decision.

[47] Yuan SS, Rehnberg C, Sun XJ (2014). Income related inequalities in new cooperative medical scheme: a five-year empirical study of Junan County in China. Int J Equity Health.

[48] Xu YJ, Zhang T, Wang DL (2019). Changes in inequality in utilization of preventive care services: evidence on China's 2009 and 2015 health system reform. Int J Equity in Health.

[49] Li CF, Dou L, Wang HP (2017). Horizontal inequity in health care utilization among the middle-aged and elderly in China. Int J Environ Res Public Health.

[50] Fu XZ, Sun N, Xu F (2018). Influencing factors of inequity in health services utilization among the elderly in China. Int J Equity Health.

[51] Fan XJ, Su M, Si YF (2021). The benefits of an integrated social medical insurance for health services utilization in rural China: evidence from the China health and retirement longitudinal study. Int J Equity Health..

[52] Liu Y, Liu N, Cheng MJ (2021). The changes in socioeconomic inequalities and inequities in health services utilization among patients with hypertension in Pearl River Delta of China, 2015 and 2019. BMC Public Health.

[53] Guo B, Xie X, Wu QH (2020). Inequality in the health services utilization in rural and urban china: a horizontal inequality analysis. Medicine.

[54] Mu LJ, Kringos DS, Kunst AE (2019). The evolution of income-related inequalities in healthcare utilization in Indonesia, 1993–2014. PLoS One.

[55] Luo JH, Zhang XL, Jin CG (2009). Inequality of access to health care among the urban elderly in northwestern China. Health Policy.

[56] Yang J, Gao M (2018). The impact of education expansion on wage inequality. Appl Econ.

[57] Shang XP, Huang YM, Li BE (2019). Residents’ awareness of family doctor contract services, status of contract with a family doctor, and contract service needs in Zhejiang Province, China: a cross-sectional study. Int J Environ Res Public Health.

[58] Zhao YR, Lin JF, Qiu YW (2017). Demand and signing of general practitioner contract service among the urban elderly: a population-based analysis in Zhejiang Province, China. Int J Environ Res Public Health.

[59] Wang C, Yan SJ, Jiang H (2021). Residents' willingness to maintain contracts with family doctors: a cross-sectional study in China. J Gen Intern Med.

[60] Gao J, Raven JH, Tang SL (2007). Hospitalisation among the elderly in urban China. Health Policy.

[61] Yan XL, Liu YL, Cai M (2022). Trends in disparities in healthcare utilization between and within health insurances in China between 2008 and 2018: a repeated cross-sectional study. Int J Equity Health.

[62] Dong ZY, Zhao CX (2020). "New medical reform" decade: my country's medical and health development achievements difficulties and path choices. Reform.

[63] Wang WJ, Cao XY (2016). Can increasing the supply of medical resources solve the problem of "expensive medical treatment"?——based on the analysis of China's inter-provincial panel data. J Manage World.

[64] Multidimensional Publicness and Collaboration for Community Benefits (2020). The case of US hospitals. Public Adm Rev.

[65] Zou H, Liu YP (2016). Heterogeneous medical insurance, out-of-pocket medical expenditure and the health level of middle-aged and middle-aged and elderly people. Finance and Economics.

